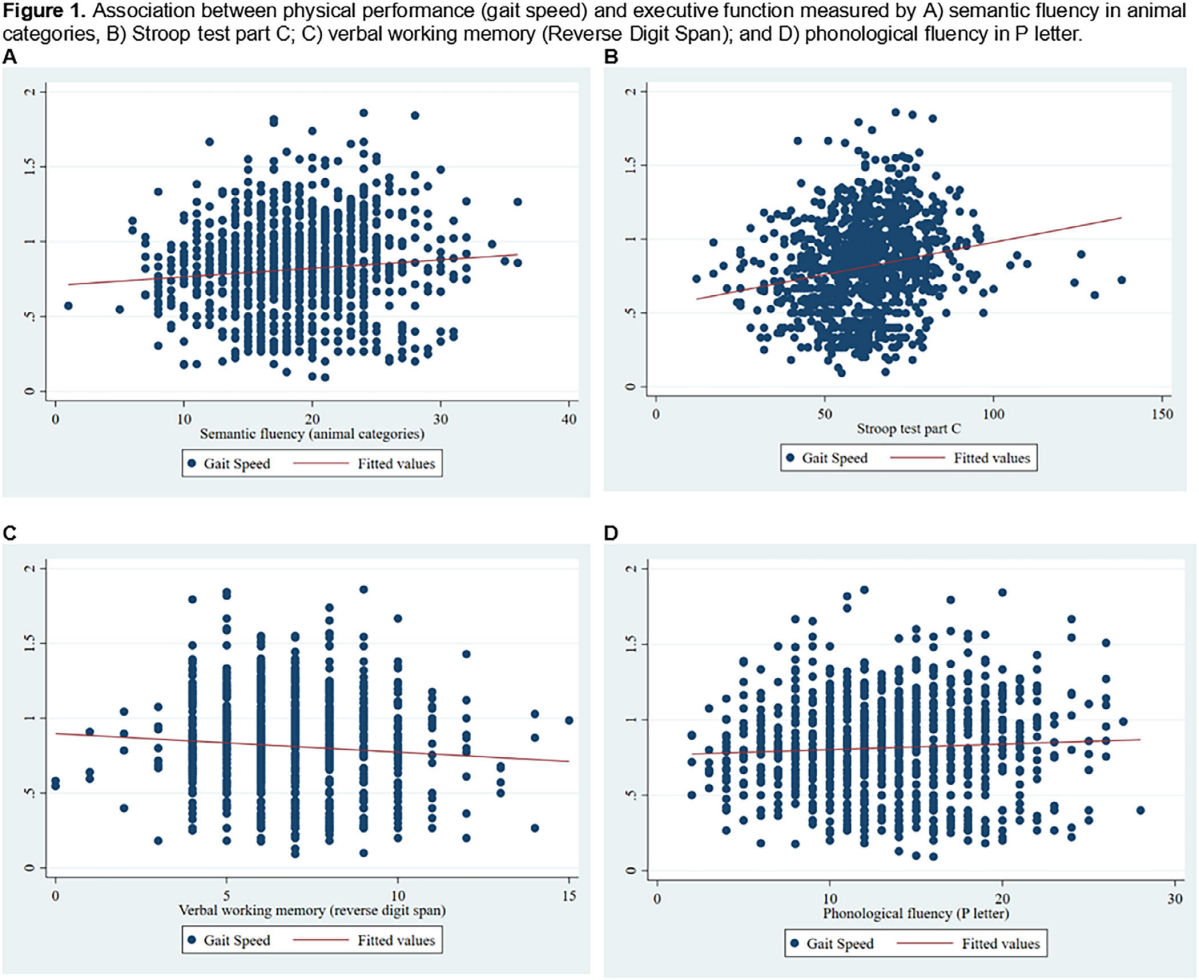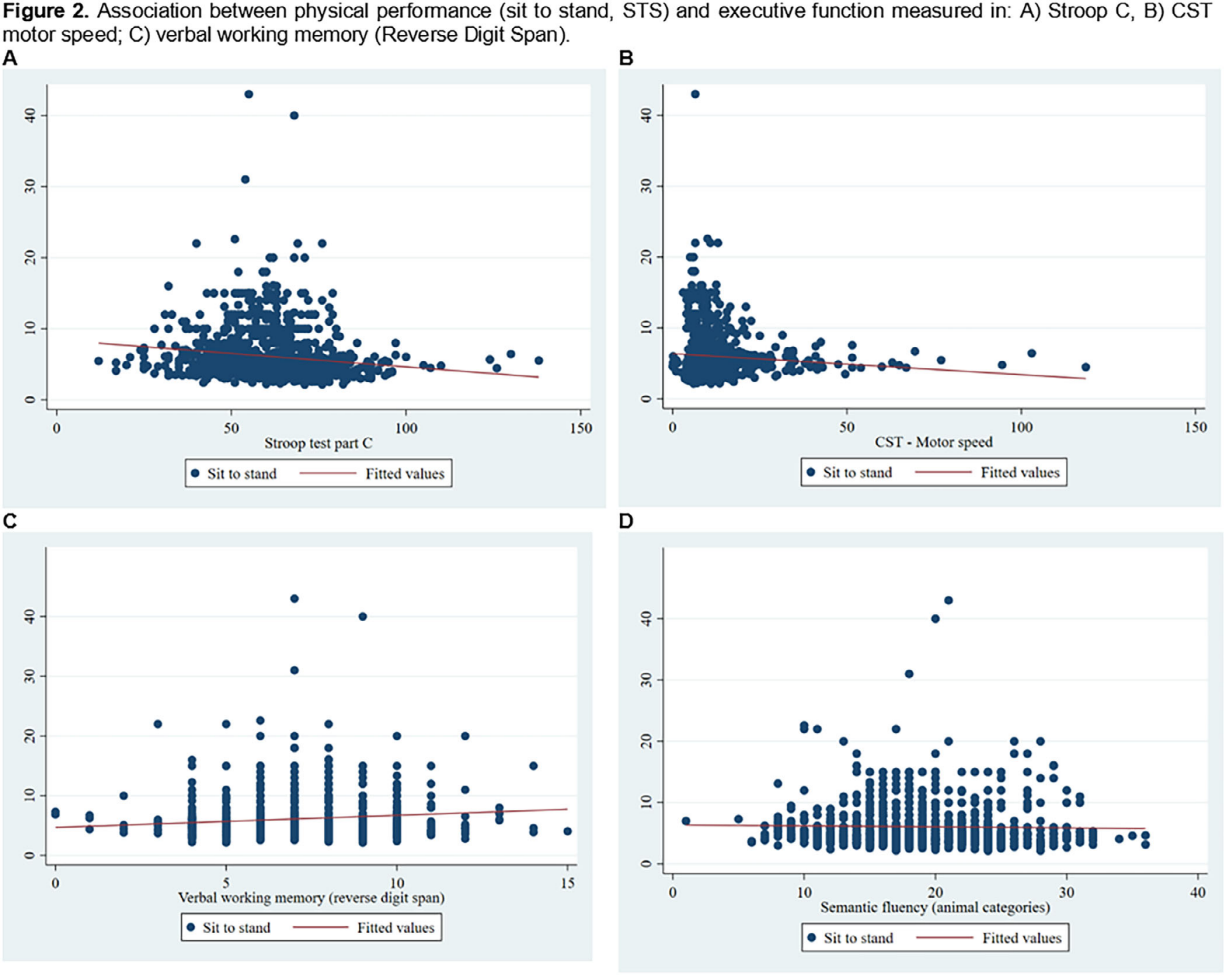# Physical Performance and its Association with Executive Function in Latin American Older Adults: Evidence from the LatAmFINGERS Multicenter Randomized Controlled Trial

**DOI:** 10.1002/alz70863_110633

**Published:** 2025-12-23

**Authors:** Myriam Gutiérrez, Alejandra Marroig, Nicole Concha, Carolina Delgado, Ana Charamelo, Pablo Burgos, Andrea Slachevsky, Sergio Dansilio, Jose Lema, Melissa Martinez, Carlos Márquez, Natalia Pozo Castro, Maira Moreno, Jacinta Bravo, Oriana Lara, Jamileth More, Natalia Acosta‐Baena, Luc¡a Crivelli, Ismael Luis Calandri, Gustavo Sevlever, David Aguillon, Ricardo Allegri, Paulo Caramelli, Ricardo Nitrini, Ana Luisa Sosa‐Ortiz, Rosa Maria Salinas‐Contreras, Claudia Kimie Suemoto, Mônica Sanches Yassuda

**Affiliations:** ^1^ Universidad de Chile, Departamento de Neurociencia, Independencia, Santiago, Santiago Chile; ^2^ Universidad Mayor, Facultad de Medicina y Ciencias de la Salud, Núcleo Magíster en Salud de la Mujer, Providencia, Santiago, Santiago Chile; ^3^ Hospital Clínico Universidad de Chile, Unidad de Cerebro Saludable, Independencia, Santiago, Santiago Chile; ^4^ Universidad Diego Portales, Escuela de Kinesiología, Santiago Centro, Santiago Chile; ^5^ Instituto de Estadística, Universidad de la República, Uruguay, Montevideo, Montevideo Uruguay; ^6^ Universidad Mayor, Facultad de Medicina y Ciencias de la Salud, Magíster en Kinesiología Gerontológica, Santiago, Santiago Chile; ^7^ Universidad de Chile, Santiago, Santiago Chile; ^8^ Hospital Clínico Universidad de Chile, Departamento de Neurología y Neurocirugía, Santiago, Santiago Chile; ^9^ Hospital Británico, Clínica de Memoria, Montevideo, Montevideo Uruguay; ^10^ Universidad de Chile, Departamento de Kinesiología, Santiago, Santiago Chile; ^11^ Oregon Health and Science University, Portland, OR USA; ^12^ Neurology Service, Department of Medicine, Clínica Alemana, Universidad del Desarrollo, Santiago, Región Metropolitana de Santiago Chile; ^13^ Cognitive Neurology and Dementia, Neurology Department, Hospital del Salvador, Santiago Chile; ^14^ Memory and Neuropsychiatric Clinic (CMYN), Neurology Service, Hospital del Salvador and Faculty of Medicine, Universidad de Chile, Santiago Chile; ^15^ Geroscience Center for Brain Health and Metabolism (GERO), Santiago, Región Metropolitana de Santiago Chile; ^16^ Neuropsychology and Clinical Neuroscience Laboratory (LANNEC), Physiopathology Department ‐ ICBM, Neuroscience and East Neuroscience Departments, Faculty of Medicine, University of Chile, Santiago Chile; ^17^ Hospital de Clínicas, Montevideo, Montevideo Uruguay; ^18^ Hospital Clínico Universidad de Chile, Unidad de Cerebro Saludable, Santiago, Metropolitana Chile; ^19^ Geroscience Center for Brain Health and Metabolism (GERO), Santiago, Region Metropolitana Chile; ^20^ Hospital Clínico Universidad de Chile, Departamento de Neurología y Neurocirugía, Santiago Chile; ^21^ Universidad de la Frontera, Departamento de Medicina Interna, Temuco, Temuco Chile; ^22^ Universidad de Chile, Instituto de Nutrición y Tecnología de los Alimentos (INTA), Unidad de Nutrición Pública, Santiago, Santiago Chile; ^23^ Hospital San Borja Arriarán, Santiago, Region Metropolitana Chile; ^24^ Global Brain Health Institute, Memory and Aging Center, University of California San Francisco, San Francisco, CA USA; ^25^ University of Chile, Santiago, Santiago Chile; ^26^ Hospital Clínico Universidad de Chile, Unidad de Cerebro Saludable, Santiago Chile; ^27^ Hospital Clínico Universidad de Chile, Unidad de Cerebro Saludable, Santiago, Santiago Chile; ^28^ Universidad de Chile, Departamento de Kinesiología, Santiago, Metropolitana Chile; ^29^ Hospital Clínico Universidad de Chile, Centro de Investigación Clínica Avanzada (CICA), Santiago, Santiago Chile; ^30^ Grupo de Genética Molecular of University of Antioquia, Medellin Colombia; ^31^ Grupo de Neurociencias de Antioquia, Facultad de Medicina, Universidad de Antioquia, Medellín, Antioquia Colombia; ^32^ Grupo de Neurociencias de Antioquia, Facultad de Medicina, Universidad de Antioquia, Medellin Colombia; ^33^ Institute of Neurosciences (INEU), Fleni‐CONICET, Buenos Aires, Buenos Aires Argentina; ^34^ Fleni, Buenos Aires Argentina; ^35^ Fleni, Buenos Aires, Buenos Aires Argentina; ^36^ Fleni, CABA, Buenos Aires Argentina; ^37^ Neurosciences Group of Antioquia, University of Antioquia, Medellín Colombia; ^38^ Faculty of Medicine ‐ Federal University of Minas Gerais, Belo Horizonte, Minas Gerais Brazil; ^39^ University of São Paulo Medical School, São Paulo, São Paulo Brazil; ^40^ Cognitive and Behavioral Neurology Unit ‐ University of São Paulo, São Paulo Brazil; ^41^ Biobank for aging studies of the University of São Paulo Medical School, São Paulo Brazil; ^42^ Dementia Laboratory, National Institute of Neurology and Neurosurgery, Mexico City Mexico; ^43^ National Institute of Neurology and Neurosurgery, Mexico City Mexico; ^44^ National Autonomous University of Mexico, Mexico City Mexico; ^45^ Dementias Laboratory, National Institute of Neurology and Neurosurgery, Mexico City, DF Mexico; ^46^ Laboratorio de demencias del Instituto Nacional de Neurología y Neurocirugía Manuel Velasco Suárez, Ciudad de Mexico Mexico; ^47^ Division of Geriatrics, University of São Paulo Medical School, São Paulo, São Paulo Brazil; ^48^ USP, São Paulo, São Paulo Brazil

## Abstract

**Background:**

The accelerated aging process in low‐ and middle‐income countries (LMIC) has led to an increased prevalence of non‐communicable diseases, including dementia, a leading cause of disability in Latin America and the Caribbean (LAC). Executive and physical performance dysfunction accelerates functional decline in older adults. While physical performance measures predict cognitive decline, evidence linking gait speed and executive function remains scarce, particularly in LAC. This study examines the association between physical performance and executive function in older adults from 12 LAC countries.

**Methods:**

A cross‐sectional study was conducted with 1,243 participants aged 60–77 from the LatAm‐FINGERS initiative. Face‐to‐face assessments gathered sociodemographic, lifestyle, and health data. Physical performance was measured using the Short Physical Performance Battery (SPPB), including balance tests, a 4‐meter gait speed test, and the five‐chair sit‐to‐stand (STS) test. The Unipedal Single‐Leg Stance (USLS) test was also included. Executive and processing speed measures included neuropsychological tests: Trail Making Test B (TMT‐B), Stroop interference index (SII), semantic fluency (animal), and phonological fluency (P and M letter), alongside Concept Shifting Test motor speed (CST‐MS) and TMT‐A. Linear regression and composite scores for executive function and speed processing were computed.

**Results:**

The median sample per country was 100 participants, with a mean age of 67.46 ± 4.67 years; 67.46% were female, 56.88% of mixed ethnicity, and 35.40% sedentary. Mean education level was 12.84 ± 3.76 years. Gait speed was directly associated with Stroop C (*p* <0.00001), Stroop P (*p* = 0.001), and semantic fluency (*p* = 0.02). STS was inversely associated with Stroop C (*p* <0.00001), and CST‐MS (*p* = 0.010); and also directly associated with working memory (*p* <0.0001). Non‐significant associations were found between gait speed and CST‐MS, STS and semantic fluency, and TMT tests. Gait speed correlated with Mini‐Mental State Examination (MMSE) scores.

**Conclusions:**

Higher physical functional performance (gait speed) associated with higher cognitive level (MMSE) is correlated with better performance in executive functions (Stroop C) in older people. This study is the first effort in LAC to associate gait speed with executive tests such as Stroop C, contributing to the understanding of physical performance utility for future preventive and diagnostic dual‐task applications.